# Antibody Binding Alters the Characteristics and Contents of Extracellular Vesicles Released by *Histoplasma capsulatum*

**DOI:** 10.1128/mSphere.00085-15

**Published:** 2016-03-30

**Authors:** Ludmila Matos Baltazar, Ernesto S. Nakayasu, Tiago J. P. Sobreira, Hyungwon Choi, Arturo Casadevall, Leonardo Nimrichter, Joshua D. Nosanchuk

**Affiliations:** a Department of Microbiology and Immunology, Albert Einstein College of Medicine, Bronx, New York, USA; b Division of Infectious Diseases, Department of Medicine, Albert Einstein College of Medicine, Bronx, New York, USA; c Biological Sciences Division, Pacific Northwest National Laboratory, Richland, Washington, USA; d Bindley Bioscience Center, Purdue University, West Lafayette, Indiana, USA; e Saw Swee Hock School of Public Health, National University of Singapore, Singapore; f Department of Molecular Microbiology and Immunology, Johns Hopkins Bloomberg School of Public Health, Johns Hopkins University, Baltimore, Maryland, USA; g Instituto de Microbiologia Paulo de Góes, Universidade Federal do Rio de Janeiro (UFRJ), Rio de Janeiro, RJ, Brazil; Duke University Medical Center

**Keywords:** *H. capsulatum*, Hsp60, monoclonal antibodies, vesicles

## Abstract

*Histoplasma capsulatum* produces extracellular vesicles containing virulence-associated molecules capable of modulating host machinery, benefiting the pathogen. Treatment of *H. capsulatum* cells with monoclonal antibodies (MAbs) can change the outcome of infection in mice. We evaluated the sizes, enzymatic contents, and proteomic profiles of the vesicles released by fungal cells treated with either protective MAb 6B7 (IgG1) or nonprotective MAb 7B6 (IgG2b), both of which bind *H. capsulatum* heat shock protein 60 (Hsp60). Our results showed that treatment with either MAb was associated with changes in size and vesicle loading. MAb treatments reduced vesicle phosphatase and catalase activities compared to those of vesicles from untreated controls. We identified 1,125 proteins in vesicles, and 250 of these manifested differences in abundance relative to that of proteins in vesicles isolated from yeast cells exposed to Hsp60-binding MAbs, indicating that surface binding of fungal cells by MAbs modified protein loading in the vesicles. The abundance of upregulated proteins in vesicles upon MAb 7B6 treatment was 44.8% of the protein quantities in vesicles from fungal cells treated with MAb 6B7. Analysis of orthologous proteins previously identified in vesicles from other fungi showed that different ascomycete fungi have similar proteins in their extracellular milieu, many of which are associated with virulence. Our results demonstrate that antibody binding can modulate fungal cell responses, resulting in differential loading of vesicles, which could alter fungal cell susceptibility to host defenses. This finding provides additional evidence that antibody binding modulates microbial physiology and suggests a new function for specific immunoglobulins through alterations of fungal secretion.

**IMPORTANCE** Diverse fungal species release extracellular vesicles, indicating that this is a common pathway for the delivery of molecules to the extracellular space. However, there has been no study reporting the impact of antibody binding to the fungal cell on extracellular vesicle release. In the present work, we observed that treatment of *H. capsulatum* cells with Hsp60-binding MAbs significantly changed the size and cargo of extracellular vesicles, as well as the enzymatic activity of certain virulence factors, such as laccase and phosphatase. Furthermore, this finding demonstrates that antibody binding can directly impact protein loading in vesicles and fungal metabolism. Hence, this work presents a new role for antibodies in the modification of fungal physiology.

## Abstract (precis)

Diverse fungal species release extracellular vesicles, indicating that this is a common pathway for the delivery of molecules to the extracellular space. However, there has been no study reporting the impact of antibody binding to the fungal cell on extracellular vesicle release. In the present work, we observed that treatment of *H. capsulatum* cells with Hsp60-binding MAbs significantly changed the size and cargo of extracellular vesicles, as well as the enzymatic activity of certain virulence factors, such as laccase and phosphatase. Furthermore, this finding demonstrates that antibody binding can directly impact protein loading in vesicles and fungal metabolism. Hence, this work presents a new role for antibodies in the modification of fungal physiology.

## INTRODUCTION

*Histoplasma capsulatum*, a dimorphic fungus, is the etiologic agent of histoplasmosis, a systemic mycosis with a worldwide distribution. *H. capsulatum* infections are common in North America, mainly in the United States (1, 2), and are also highly prevalent in some Latin American countries, such as Brazil, Venezuela, Ecuador, Paraguay, and Argentina (3, 4). Infection occurs after inhalation of microconidia or hyphal fragments from the environment by a susceptible host, and the lung is the primary organ of infection (5, 6). Containment of the infection involves the activation of cell-mediated immunity with uptake of fungi by phagocytic cells such as neutrophils and macrophages (5, 7). Interestingly, *H. capsulatum* yeast cells subvert the intraphagosomal milieu, maintaining an environment that is permissive to fungal multiplication (5, 8). Although the role of humoral immunity in the pathogenesis of histoplasmosis is uncertain, monoclonal antibodies (MAbs) have been shown to significantly improve survival after a lethal challenge in a murine infection model (9, 10). Interestingly, we previously demonstrated that two competing MAbs to heat shock protein 60 (Hsp60) of different subtypes had dramatically different effects on disease pathogenesis, with MAb 6B7 (IgG1) producing a protective response and MAb 7B6 (IgG2b) enhancing the disease (9).

Over the past decade, several studies have shown that fungi produce extracellular vesicles. This remarkable process involves the transport of macromolecule-containing vesicles across the complex fungal cell wall, a secretory machinery that is utilized by diverse ascomycetes and basidiomycetes, including *H. capsulatum*, *Candida albicans*, *Cryptococcus neoformans*, *Malassezia sympodialis*, *Paracoccidioides brasiliensis*, and *Alternaria infectoria* (11–16). Analyses of the contents of vesicles from these different fungi have revealed the presence of lipids, phospholipids, polysaccharides, nucleic acid, proteins, and virulence factors, such as laccase and urease (11, 17, 18). In *H. capsulatum*, the extracellular vesicles contain important proteins involved in fungal pathogenesis and stress responses, including Hsp60, which suggest the participation of fungal extracellular vesicles in the establishment and progression of disease (15).

It is notable that several of the described virulence factors of *H. capsulatum* that have been identified in the secreted vesicles are unconventional cell wall components. For example, the chaperone Hsp60 is a major ligand involved in phagocytosis by mediating the attachment of *H. capsulatum* cells to macrophage/monocyte integrin CR3 (CD11b/CD18), whereas M antigen, another surface antigen, is a catalase involved in the protection of fungal cells from oxidative stress (9, 19). In addition, phosphatase and laccase are enzymes involved in protein dephosphorylation and melanin synthesis, respectively (19, 20). Given the finding that MAbs can modify disease pathogenesis, we determined the effects of a protective MAb and a nonprotective MAb on the production and contents of extracellular vesicles from *H. capsulatum*.

## RESULTS

### DLS analysis of extracellular vesicles released after treatment of *H. capsulatum* cells with a protective (6B7) or nonprotective (7B6) antibody.

**FIG 1:**
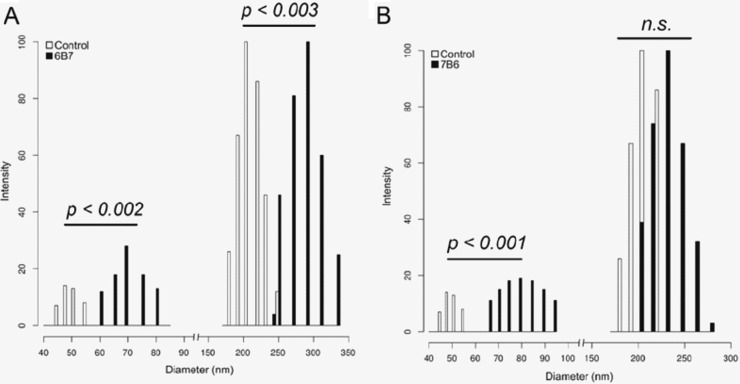
Distribution of extracellular vesicle dimensions obtained from control *H. capsulatum* yeast cells compared to vesicle size ranges obtained from yeast cells treated with MAb 6B7 (A) or 7B6 (B). Control: *H. capsulatum* cells not treated with MAbs.

Dynamic light scattering (DLS) was used to evaluate the vesicle sizes in each sample (Fig. 1A and B). The results show that incubation of *H. capsulatum* cells with MAbs 6B7 and 7B6 significantly changed the size of the vesicles released by the fungal cells in comparison with that of vesicles released by untreated yeast cells (Fig. 1A and B). Vesicles collected from untreated control cells were found to occur in two distinct size ranges: a small population varying between 40 and 60 nm and those of a larger size ranging between 170 and 250 nm in diameter. After treatment with MAb 6B7, the sizes of both vesicle populations increased compared with those of the control. The sizes of small and large vesicles ranged between 60 and 80 nm and 240 and 350 nm, respectively. Cells treated with MAb 7B6 produced small vesicles that varied between 55 and 100 nm and larger vesicles that varied between 200 and 300 nm.

### Protein and sterol content quantification after treatment of *H. capsulatum* cells with protective (6B7) and nonprotective (7B6) antibodies.

**FIG 2:**
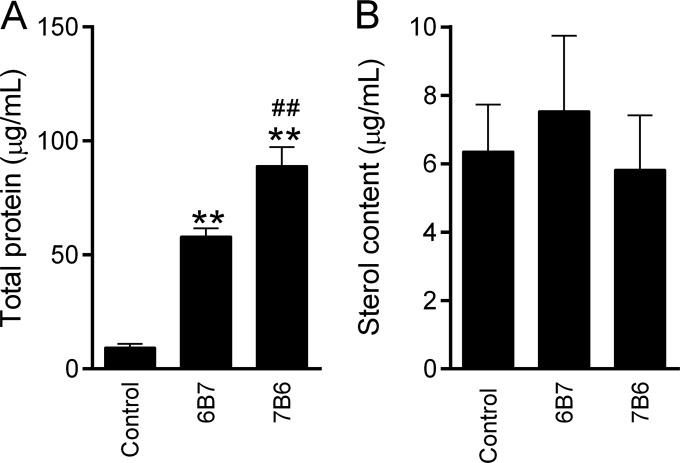
Total protein analysis and sterol content quantification in vesicles from *H. capsulatum* yeast cells with or without treatment with MAb 6B7 or 7B6. (A) Bradford assay for protein quantification. (B) Sterol content quantification. *H. capsulatum* cells were grown in Ham’s F12 medium for 7 days. The vesicles were collected and suspended in 0.5 ml of PBS. All of the analyses were performed in duplicate. **, *P* < 0.05 compared to the untreated control (*H. capsulatum* cells not treated with MAbs); ##, *P* < 0.05 compared to MAb 6B7.

The total protein and sterol concentrations of extracellular vesicles were determined with the Bradford assay and an Amplex Red kit, respectively (Fig. 2A and B; see Table S1 in the supplemental material). The structural differences between fungal and mammalian sterols do not interfere with the kit’s detection activity (14). Analysis of the total protein from extracellular vesicles shows that treatment of *H. capsulatum* with either MAb 6B7 or 7B6 results in a significant increase in protein compared with that in vesicles from untreated control yeast cells (Fig. 2A). The total protein in vesicles collected from *H. capsulatum* incubated with MAbs 6B7 and 7B6 increased 6- and 9.5-fold, respectively, in comparison with that in untreated control vesicles. In addition, the amount of protein in vesicles from *H. capsulatum* treated with MAb 7B6 was greater than that of protein in vesicles from *H. capsulatum* treated with MAb 6B7 (Fig. 2A). Interestingly, the amount of fungal sterol in the vesicles did not change after treatment with MAbs 6B7 and 7B6 compared to that in the untreated control (Fig. 2B).

10.1128/mSphere.00085-15.1 Table S1  Total protein analysis and sterol content quantification in vesicles from *H. capsulatum* yeast cells with or without treatment with MAb 6B7 or 7B6. Download Table S1, DOCX file, 0.01 MB. Copyright © 2016 Matos Baltazar et al. 2016 Matos Baltazar et al. This content is distributed under the terms of the Creative Commons Attribution 4.0 International license.

### Enzymatic assay of vesicles derived from *H. capsulatum* cells with or without MAb treatment.

**FIG 3:**
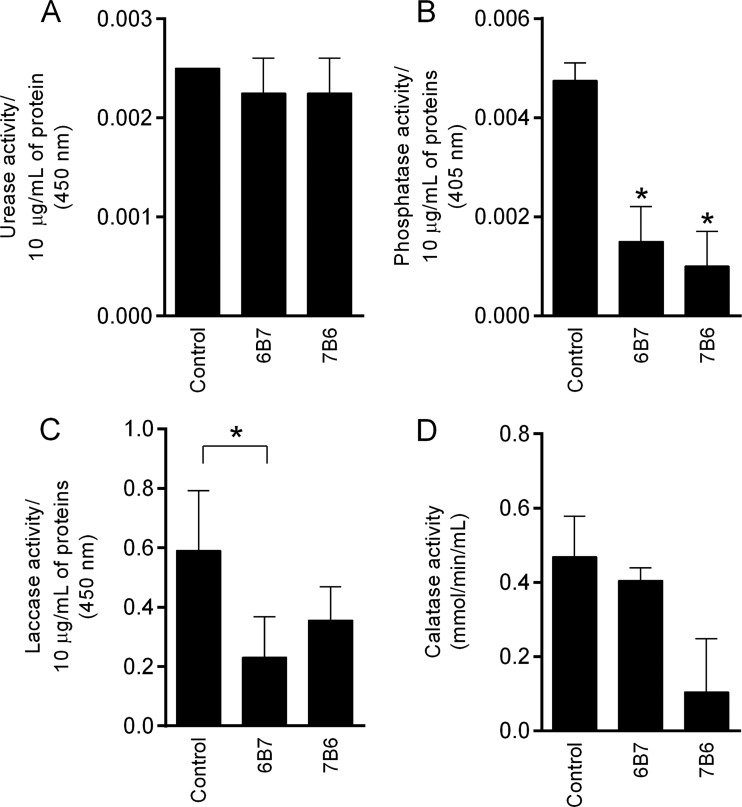
Enzymatic activities of extracellular vesicles. Urease (A), phosphatase (B), laccase (C), and catalase (D) activities were measured. All of the analyses were performed in duplicate. *, *P* < 0.05 compared to the untreated control (*H. capsulatum* cells not treated with MAbs).

To detect urease, phosphatase, laccase, and catalase activities, suspensions of vesicles were added to an enzyme reaction solution specific to each enzyme evaluated. The activities of these enzymes were detected in extracellular vesicles isolated from *H. capsulatum* yeasts with or without MAb treatment (Fig. 3A and B). Although the urease activity levels were similar in control and antibody-treated vesicles (Fig. 3A), phosphatase, laccase, and catalase activities were modified by antibody treatment (Fig. 3B to D). Both MAbs 6B7 and 7B6 significantly decreased the phosphatase activity in vesicles compared to that in the untreated control (Fig. 3B). Laccase activity was significantly lower in vesicles from cells incubated with MAb 6B7 than in either untreated control (Fig. 3C). There was also a trend toward lower laccase and catalase activities in vesicles isolated from cells treated with MAb 7B6 than in vesicles isolated from the untreated control. In addition, the catalase activity levels were similar in control and MAb 6B7-treated vesicles.

### Proteomic analysis of extracellular vesicles of *H. capsulatum* cells treated with MAb 6B7 or 7B6.

**FIG 4:**
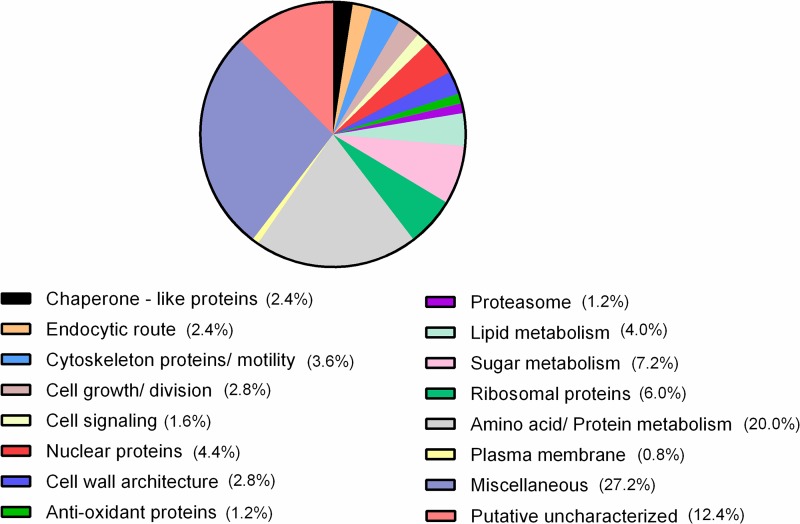
Differentially abundant proteins in vesicles isolated from *H. capsulatum* yeast cells incubated with MAb 6B7 or 7B6 compared to those in vesicles isolated from untreated yeast cells.

Protein analysis was performed after vesicle purification and enzymatic digestion. Identification of individual peptides was achieved by searching tandem mass spectra against a sequence database containing the *H. capsulatum* complete proteome set from the UniProt Knowledge Base and common contaminant sequences with the Paragon tool of the Protein Pilot software (AB Sciex). Complete proteomic analysis of the *H. capsulatum* extracellular vesicles isolated from each of the conditions examined led to the identification of a total of 1,125 proteins that were separated into 1,117 groups (see Tables S2 and S3 in the supplemental material), in which a protein group is defined by isoforms that have the same peptides. Of the 1,117 protein groups, 699 had peptide intensities above the limit of quantification (see Table S4 in the supplemental material) and 250 proteins in this subset were differentially abundant (Table 1; see Table S5 in the supplemental material). Figure 4 depicts the best-represented protein categories organized according to their biological processes. This classification based on biological processes shows that the most plentiful of the proteins are related to amino acid/protein metabolism (20%), followed by proteins associated with sugar metabolism (7.2%), nuclear proteins, and lipid metabolism (both 4%). In addition, 27.6% of the proteins were grouped together as miscellaneous and 12.4% were uncharacterized proteins.

10.1128/mSphere.00085-15.2 Table S2  Identified peptides and proteins. Download Table S2, XLSX file, 1.2 MB. Copyright © 2016 Matos Baltazar et al. 2016 Matos Baltazar et al. This content is distributed under the terms of the Creative Commons Attribution 4.0 International license.

10.1128/mSphere.00085-15.3 Table S3  Identified proteins and protein groups. Download Table S3, XLSX file, 0.2 MB. Copyright © 2016 Matos Baltazar et al. 2016 Matos Baltazar et al. This content is distributed under the terms of the Creative Commons Attribution 4.0 International license.

10.1128/mSphere.00085-15.4 Table S4  Quantitative proteomic analysis. Download Table S4, XLSX file, 0.1 MB. Copyright © 2016 Matos Baltazar et al. 2016 Matos Baltazar et al. This content is distributed under the terms of the Creative Commons Attribution 4.0 International license.

10.1128/mSphere.00085-15.5 Table S5  Differentially abundant proteins. Download Table S5, XLSX file, 0.1 MB. Copyright © 2016 Matos Baltazar et al. 2016 Matos Baltazar et al. This content is distributed under the terms of the Creative Commons Attribution 4.0 International license.

**TABLE 1:** Details of the differentially abundant proteins found in *H. capsulatum* vesicles

Protein type and hit no.	Accession no.	Identification	Function
Chaperone-like proteins			
1	C0NEZ9	Receptor-associated protein	Intracellular protein transport
2	C0NS16	DnaK-type molecular chaperone BipA	Chaperone
3	C0NBV8	Heat shock protein	Chaperone
4	C0NYC6	Hsp70-like protein	Chaperone
5	C0P0B3	Hsp60-like protein	Chaperone
6	C0P152	Heat shock protein	Chaperone
Endocytic-route proteins			
7	C0NI41^b^	VHS domain-containing protein	Intracellular protein transport
8	C0NKH9^a^	ADP-ribosylation factor	GTP binding
9	C0NA79^a^	Prenylated Rab accepter 1	Involved in transport between ER^d^ and Golgi complex
10	C0NJL9	Vacuolar-sorting-associated protein	Vesicular protein sorting
11	C0NXJ2	Secretory pathway GDP dissociation inhibitor	Rab GDP-dissociation inhibitor activity
12	C0NRE5^a^	ε-COP	Retrograde vesicle-mediated transport, Golgi to ER
Cytoskeleton/motility proteins			
13	C0P0B4	Cofilin	Actin binding
14	C0NA44	Coronin	Actin-associated protein
15	C0NBZ7	F-actin-capping protein subunit β	Actin binding
16	C0NMF2	Fimbrin	Protein binding
17	C0NTH2^a^	Septin	Cytokinesis
18	P53455	Actin	Cytoskeleton assembly
19	C0NKB3	Tubulin β chain	Structural constituent of cytoskeleton
20	C0P0S2	Tubulin α chain	Structural constituent of cytoskeleton
21	C0P074	Tubulin α-1 subunit	Structural constituent of cytoskeleton
Cell growth/division proteins			
22	C0NBG1	DNA damage checkpoint protein Rad24	DNA damage checkpoint
23	C0NC23	dUTPase	
24	C0NFW3^a^	RNA polymerase Rpb1 C-terminal repeat domain-containing protein	Transcription of DNA
25	C0NQN9	Septin	GTP binding
26	C0NF61	Cell division control protein	ATP binding
27	C0NXU1	Flap endonuclease 1	DNA binding
28	C0NML9^b^	Mitogen-activated protein kinase	MAP kinase activity
Cell signaling proteins			
29	C0NFM9^b^	Small G-β protein GPB	Protein binding
30	C0NFN5^b^	Ran-specific GTPase-activating protein	Intracellular transport
31	C0NIC1^a^	PH domain-containing protein	Intracellular signaling
32	C0P083	GTP-binding protein ypt3	Small GTPase-mediated signal transduction
Nuclear proteins			
33	C0NCI0	Uracil-DNA glycosylase	Uracil DNA *N-*glycosylase activity
34	C0NRN4^b^	Histone H2A	
35	C0NZ94	Histone H2B	DNA binding
36	C0NL60	Histone H3	DNA binding
37	C0P057^b^	RuvB-like helicase	DNA helicase activity
38	C0NJZ2^a^	RuvB-like helicase 1	DNA helicase activity
39	C0P170^a^	Cap-binding protein	RNA metabolic process
40	C0NQX1	DNA ATP-dependent helicase	DNA binding, ATP binding
41	C0NPP2	DNA damage-binding protein 1a	Nucleic acid binding
42	C0NFM3^b^	XPG I region protein	DNA repair
43	C0P0I5	Woronin body major protein	Translation elongation factor activity
Cell wall architecture			
44	C0ND43	Cell wall synthesis protein	Cell wall synthesis
45	C0NLL2	Glucanosyltransferase	Cell wall assembly
46	C0NKE9	β-Glucosidase	Carbohydrate metabolic process
47	C0NW75	Chitinase	Chitinase activity
48	C0NSG6	Extracellular cell wall glucanase Crf1	Hydrolase activity, hydrolyzing O-glycosyl compounds
49	C0NH39	1,3-β-Glucanosyltransferase	Carbohydrate metabolic process
50	C0NIP3	GPI-anchored cell wall organization protein Ecm33	
Antioxidant proteins			
51	C0NAP3	Polyphenoloxidase	Oxidoreductase activity
52	C0NI23	Glutathione peroxidase	Glutathione peroxidase activity
53	C0NMI3	Thiol-specific antioxidant	Antioxidant activity
Proteasome proteins			
54	C0NV29^a^	Proteasome subunit α type	Endopeptidase activity
55	C0P150	Proteasome subunit β type	Endopeptidase activity
56	C0NYE5	26S proteasome regulatory subunit	Protein binding
Lipid metabolism proteins			
57	C0NMK6^b^	Acyl-CoA^c^ dehydrogenase	Acyl-CoA dehydrogenase activity
58	C0NNM0	3-Ketoacyl-CoA thiolase	Catalytic activity
59	C0NJW7	3-Ketoacyl-CoA thiolase peroxisomal A	Catalytic activity
60	C0NY84	Glycerophosphoryl diester phosphodiesterase	Glycerol metabolic process
61	C0NZL5	Enoyl-CoA hydratase/isomerase	Catalytic activity
62	C0NLE5	δ-9 fatty acid desaturase	Insertion of double bond at δ position of fatty acids
63	C0P0B7	Long-chain fatty acid CoA ligase	Catalytic activity
64	C0NUX2^a^	Oxysterol-binding protein	Ergosterol synthesis
65	C0NAZ6^a^	Oxysterol-binding protein	Ergosterol synthesis
66	C0NTW1	Oxidosqualene:lanosterol cyclase	Intramolecular transferase activity
Sugar metabolism proteins			
67	C0NIP4^b^	*N*-Glycosyltransferase	Catalysis of glycosyl group transfer
68	C0P090	Citrate synthase	Citrate (Si)-synthase activity
69	C0NHJ7	Glucosidase I	Mannosyl-oligosaccharide glucosidase activity, catalytic activity
70	C0NGE0	Aconitate hydratase	Tricarboxylic acid cycle
71	C0NRA2	Sugar transporter	Transporter activity
72	C0NJB1	Phosphoglycerate kinase	Phosphoglycerate kinase activity
73	C0NRR6	Ribose 5-phosphate isomerase A	Ribose-5-phosphate isomerase activity
74	C0NUY1	Fructose 1,6-biphosphate aldolase	Fructose-bisphosphate aldolase activity
75	C0NQI1	Fructose-1,6-bisphosphatase	Fructose 1,6-bisphosphate 1-phosphatase activity
76	C0NDI4	β-Glucosidase	Carbohydrate metabolic process
77	C0NFD3	Triosephosphate isomerase	Glycolytic process
78	C0NRN1	Glyceraldehyde-3-phosphate dehydrogenase	Glucose metabolic process
79	C0P046	Malate dehydrogenase	Malate metabolic process
80	C0NDH1	Malate dehydrogenase	Malate metabolic process
81	C0NH60	Aconitase	Tricarboxylic acid cycle
82	C0NAG1	Pyruvate carboxylase	Pyruvate metabolic process
83	C0NV40	*N*-Acetylglucosamine-phosphate mutase	Carbohydrate metabolic process
84	C0NHP7	Isocitrate lyase	Isocitrate lyase activity
Ribosomal proteins			
85	C0NC89^a^	60S acidic ribosomal protein P0	Structural constituent of ribosome
86	C0NE75	60S ribosomal protein L23	Structural constituent of ribosome
87	C0NKI2	60S ribosomal protein L1	Structural constituent of ribosome
88	C0NLP3	40S ribosomal protein S4	Structural constituent of ribosome
89	C0NMA2	Ribosomal protein L19	Structural constituent of ribosome
90	C0NRD6	60S ribosomal protein L5	Structural constituent of ribosome
91	C0NUD0	40S ribosomal protein S3	Structural constituent of ribosome
92	C0NUE8^a^	40S ribosomal protein S12	Structural constituent of ribosome
93	C0NHN4^a^	Ribosomal protein L14	
94	C0NYP9	60S ribosomal protein L13	Structural constituent of ribosome
95	C0NKW7	40S ribosomal protein S0	Structural constituent of ribosome
96	C0NDC6	Large-subunit ribosomal protein L3	Structural constituent of ribosome
97	C0NCE3^b^	60S ribosomal protein L20	Structural constituent of ribosome
98	C0NRH5	60S ribosomal protein L24	Structural constituent of ribosome
99	C0NVC6	40S ribosomal protein S17	Structural constituent of ribosome
Amino acids/proteins involved in metabolism			
100	C0NUQ8	Probable dipeptidyl-aminopeptidase B	Serine-type peptidase activity
101	C0NIM4	Glutamate dehydrogenase	Cellular amino acid metabolic process
102	C0NXA3	Eukaryotic translation initiation factor 3 subunit C	Translation initiation factor activity
103	C0NAB3	Probable carboxypeptidase HCBG_00059	Hydrolase activity
104	C0NAK7	Aspartyl aminopeptidase	Aminopeptidase activity
105	C0NAW2^b^	Eukaryotic translation initiation factor 3 subunit I	Protein synthesis
106	C0NCH9	Protein disulfide-isomerase	Protein folding
107	C0NEA1	Fumarylacetoacetase	Aromatic amino acid family metabolic process
108	C0NIW4	Cobalamin-independent methionine synthase MetH/D	Methionine biosynthetic process
109	C0NKL7	Peptidyl-prolyl *cis*-*trans* isomerase	Peptidyl-prolyl *cis*-*trans* isomerase activity
110	C0NNC2^b^	Aminopeptidase	Metallopeptidase activity
111	C0P0D5	Aminopeptidase	Metallopeptidase activity
112	C0NND5	Thioredoxin	Protein disulfide oxidoreductase activity
113	C0NJC3	Adenosylhomocysteinase	Adenosylhomocysteinase activity
114	C0NQ08	Saccharopine dehydrogenase [NAD^(+)^, l-lysine forming]	Saccharopine dehydrogenase (NAD^+^, l-lysine-forming) activity
115	C0NRP4	Peptidyl-prolyl *cis*-*trans* isomerase	Peptidyl-prolyl *cis*-*trans* isomerase activity
116	C0NSI9^a^	Carboxypeptidase	Serine-type carboxypeptidase activity
117	C0NSN4	Elongation factor 2	GTP binding, GTPase activity
118	C0NSU0	Serine/threonine phosphatase	Hydrolase activity
119	C0NT48^a^	A-pheromone-processing metallopeptidase Ste23	Catalytic activity, metal ion binding
120	C0NUD2	Phosphoprotein phosphatase A	Binding
121	C0NNE2	3-Isopropylmalate dehydratase	Leucine biosynthetic process
122	C0NVD3	Kynureninase	Kynureninase activity
123	C0NVD9^a^	Eukaryotic translation initiation factor 3 subunit L	Eukaryotic translation initiation factor 3 complex
124	C0NBB3	Peptidyl-prolyl *cis*-*trans* isomerase	
125	C0NE91	Seryl-tRNA synthetase	
126	C0NJ54	Elongation factor Tu	
127	C0NPC9	Ubiquitin-activating enzyme	Small-protein-activating enzyme activity
128	C0NVW8	Peptidyl-prolyl *cis*-*trans* isomerase	Protein folding
129	C0NXH6^b^	Ornithine aminotransferase	Pyridoxal phosphate binding
130	C0NXL8^b^	Argininosuccinate lyase	Arginine biosynthetic process via ornithine
131	C0NZA7	Cytosolic nonspecific dipeptidase	Hydrolase activity
132	C0NZE4	d-Tyrosyl-tRNA(Tyr) deacylase	
133	C0NN56	Glutamine synthetase	Glutamate-ammonia ligase activity
134	P40911	Elongation factor 1-α	Translation elongation factor activity during protein biosynthesis
135	C0NAN2	ATP-dependent RNA helicase EIF4A	Nucleic acid binding
136	C0NZL2	Ketol-acid reductoisomerase	Branched-chain amino acid biosynthetic process
137	C0NEM8^a^	3-Isopropylmalate dehydrogenase	Leucine biosynthetic process
138	C0NX46^a^	Carboxypeptidase Y homolog A	Serine-type carboxypeptidase activity
139	C0NL66	Isoleucyl-tRNA synthetase, cytoplasmic	Aminoacyl-tRNA ligase activity
140	C0NJU5^a^	Saccharopine dehydrogenase	Oxidoreductase activity
141	C0NBP4^a^	Aromatic amino acid aminotransferase	Pyridoxal phosphate binding
142	C0NGY7	Aspartyl-tRNA synthetase	tRNA aminoacylation for protein translation
143	C0NLE3	Metallopeptidase MepB	Metalloendopeptidase activity
144	C0NXZ3	Serine/threonine-protein kinase DCLK1	Protein phosphorylation
145	C0NQD6	Phospho-2-dehydro-3-deoxyheptonate aldolase	Aromatic amino acid family biosynthetic process
146	C0NBP7^a^	Calcium/calmodulin-dependent protein kinase	Protein phosphorylation
147	C0NFN7^b^	α-1,2-Mannosyltransferase Kre5	Mannosyltransferase activity
148	C0NV96^b^	Phospho-2-dehydro-3-deoxyheptonate aldolase	Aromatic amino acid family biosynthetic process
Plasma membrane proteins			
149	C0P028	DUF895 domain-containing protein	Transmembrane transport
150	C0P096	Plasma membrane ATPase	ATP biosynthetic process
Miscellaneous proteins			
151	C0NDZ9	Pyridoxine biosynthesis protein PyroA	Pyridoxal phosphate biosynthetic process
152	C0NDZ7	Probable Xaa-Pro aminopeptidase P	Hydrolase activity
153	C0NB22^b^	RNA-binding protein	Nucleotide binding
154	C0NB64^b^	Short-chain dehydrogenase/reductase	Oxidoreductase activity
155	C0NBU6	Esterase	*S*-Formylglutathione hydrolase activity
156	C0NBV1^a^	Cyclin-dependent protein kinase PhoA	Transferase activity, transferring phosphorus-containing groups
157	C0NCA6^a^	Serine/threonine-protein phosphatase	Hydrolase activity
158	C0NCC8^a^	Phosphatase PP1 regulatory subunit sds22	Protein binding
159	C0NQI2	Fumarate reductase flavoprotein subunit	Succinate dehydrogenase activity
160	C0NEV9^b^	Fibrillarin	RNA binding
161	C0NFD1^b^	Armadillo repeat protein	Protein binding
162	C0NG75	ATP synthase subunit α	ATP binding
163	C0NIZ7^b^	Nicotinate-nucleotide pyrophosphorylase (carboxylating)	NAD biosynthetic process
164	C0NHZ2^b^	Prohibitin	DNA synthesis inhibition
165	C0NJV2	Aha1 domain family	Chaperone binding
166	C0NJV7	V-type proton ATPase subunit A	Hydrogen ion transmembrane transporter activity
167	C0NLZ4	Isochorismatase domain-containing protein	Catalytic activity
168	C0P0E1	NADH-ubiquinone oxidoreductase	ATP synthesis-coupled electron transport
169	C0NP32^a^	DUF221 domain-containing protein	
170	C0NP52	Cytochrome *b*-*c*_1_ complex subunit Rieske, mitochondrial	Ubiquinol-cytochrome *c* reductase activity, oxidoreductase activity
171	C0NQQ6^a^	MYG1 protein	
172	C0NQX6	Alcohol dehydrogenase	Oxidoreductase activity
173	C0NR06^b^	Adenosine kinase	Adenosine kinase activity
174	C0NRW1	Endonuclease/exonuclease/phosphatase	
175	C0NT49	Cleavage- and polyadenylation-specific factor 5	mRNA binding
176	C0NSJ0	Vacuolar ATP synthase subunit B	ATP hydrolysis-coupled proton transport
177	C0NTN5	Nucleoside diphosphate kinase	Nucleoside diphosphate kinase activity
178	C0NUW2	Hydroxymethylglutaryl-CoA synthase	Hydroxymethylglutaryl-CoA synthase activity
179	C0NVG5	Sterigmatocystin 8-*O*-methyltransferase	*O*-Methyltransferase activity
180	C0NAB0	Enolase	Phosphoenolpyruvate hydratase activity
181	C0NFJ2^b^	Oxidoreductase	Oxidoreductase activity
182	C0NLK3^b^	Glutathione-dependent formaldehyde dehydrogenase	
183	C0NSW3	Aldehyde dehydrogenase	
184	C0NTQ2	CRAL/TRIO domain-containing protein	
185	C0NHP0	2-Methylcitrate dehydratase	2-Methylcitrate dehydratase activity
186	C0NX78	Phosphoribosylformylglycinamidine cyclo-ligase	Purine nucleobase biosynthetic process
187	C0NYQ7^a^	Xanthine phosphoribosyltransferase	Nucleoside metabolic process
188	C0NZ16^a^	FAD^e^-dependent oxidoreductase superfamily	Oxidoreductase activity
189	C0NZ33	Choline sulfatase	Sulfuric ester hydrolase activity
190	C0P037^b^	NAD^+^-dependent betaine aldehyde dehydrogenase	Oxidoreductase activity
191	C0P0C5	ATP synthase subunit β	ATP binding
192	C0P0H6	3-Methylcrotonyl-CoA carboxylase biotin-containing subunit	Biotin carboxylase activity
193	C0NTZ5	RNA-binding protein Snd1	Transcription cofactor activity
194	C0NI02	Transketolase	Transketolase activity
195	C0P141	Allergen Aspf4	Allergen
196	C0NCZ0	Carnitine acetyltransferase	Transferase activity, transferring acyl groups
197	C0NP90	Alkaline phosphatase	Phosphatase activity
198	C0NBI7	Alkaline phosphatase	Phosphatase activity
199	C0P0T5^b^	NADH-ubiquinone oxidoreductase	
200	C0NTA4	Farnesyl-pyrophosphate synthetase	Isoprenoid biosynthetic process
201	C0NN26	Calnexin	Calcium ion binding
202	C0NUH0	KH domain RNA-binding protein	Nucleic acid binding
203	C0NC54	ATP synthase subunit gamma	ATP synthesis-coupled proton transport
204	C0NHD9^b^	Amidohydrolase	Nitrogen compound metabolic process
205	C0NZZ4^a^	2-Nitropropane dioxygenase	Nitronate monooxygenase activity
206	C0NC22^a^	Pyridoxine kinase	Pyridoxal kinase activity
207	C0NKA1	Vacuolar ATP synthase subunit C	ATP hydrolysis-coupled proton transport
208	C0NQK3	Adenylosuccinate lyase	Purine ribonucleotide biosynthetic process
209	C0NTY6^b^	Serine/threonine-protein phosphatase	Hydrolase activity
210	C0NW72^a^	RNase T2-like protein	RNase T2 activity
211	C0NCF6	NADP-dependent mannitol dehydrogenase	Oxidoreductase activity
212	C0NNA3	DUF757 domain-containing protein	
213	C0NK86^b^	DUF255 domain-containing protein	Catalytic activity
214	C0NFK8	Ribonucleotide reductase M2 B	Deoxyribonucleoside diphosphate metabolic process
215	C0NP11	Golgi apyrase	Hydrolase activity
216	C0NZS2	MBOAT family protein	
217	C0NMY3	Ubiquitin	Protein binding
218	C0NB50^a^	Indoleamine 2,3-dioxygenase	Heme binding
219	C0NZM7	S import receptor	Intracellular protein transport
Putative uncharacterized proteins			
220	C0NBY6	Putative uncharacterized protein	
221	C0NA95	Putative uncharacterized protein	
222	C0NBS0^a^	Putative uncharacterized protein	
223	C0NUB2	Putative uncharacterized protein	
224	C0NVK4	Putative uncharacterized protein	
225	C0NA87^b^	Putative uncharacterized protein	
226	C0NH90	Putative uncharacterized protein	Catalytic activity
227	C0NIF7	Putative uncharacterized protein	
228	C0NIS9	Putative uncharacterized protein	Endo-DNase activity, producing 5′-phosphomonoesters
229	C0NJD6	Putative uncharacterized protein	Protein binding
230	C0P165^a^	Putative uncharacterized protein	Protein binding
231	C0NK83	Putative uncharacterized protein	
232	C0NKI6^a^	Putative uncharacterized protein	
233	C0NLZ9	Putative uncharacterized protein	
234	C0NGJ6^a^	Putative uncharacterized protein	
235	C0NNW9^a^	Putative uncharacterized protein	Endocytosis
236	C0NQ22	Putative uncharacterized protein	
237	C0NRU5^b^	Putative uncharacterized protein	Protein binding
238	C0NAA6	Putative uncharacterized protein	
239	C0NAT6	Putative uncharacterized protein	
240	C0NF33^a^	Putative uncharacterized protein	
241	C0NH51^a^	Putative uncharacterized protein	
242	C0NSF6	Putative uncharacterized protein	
243	C0NST1^a^	Putative uncharacterized protein	
244	C0NUK9	Putative uncharacterized protein	
245	C0NTJ9	Putative uncharacterized protein	Transport
246	C0P1A8	Putative uncharacterized protein	Structural constituent of ribosome
247	C0P1C6^a^	Putative uncharacterized protein	Carbohydrate metabolic process
248	C0ND33	Putative uncharacterized protein	
249	C0NNS2^b^	Putative uncharacterized protein	Nucleic acid binding
250	C0NW09	Putative uncharacterized protein	Protein binding

^a^ Protein found in the *H. capsulatum* vesicles only after treatment of *H. capsulatum* with MAb 6B7.

^b^ Protein found in the *H. capsulatum* vesicles only after treatment of *H. capsulatum* with MAb 7B6.

^c^ CoA, coenzyme A.

^d^ ER, endoplasmic reticulum.

^e^ FAD, flavin adenine dinucleotide.

### Comparison of the proteomes of vesicles of *H. capsulatum* cells treated with MAbs 6B7 and 7B6.

**FIG 5:**
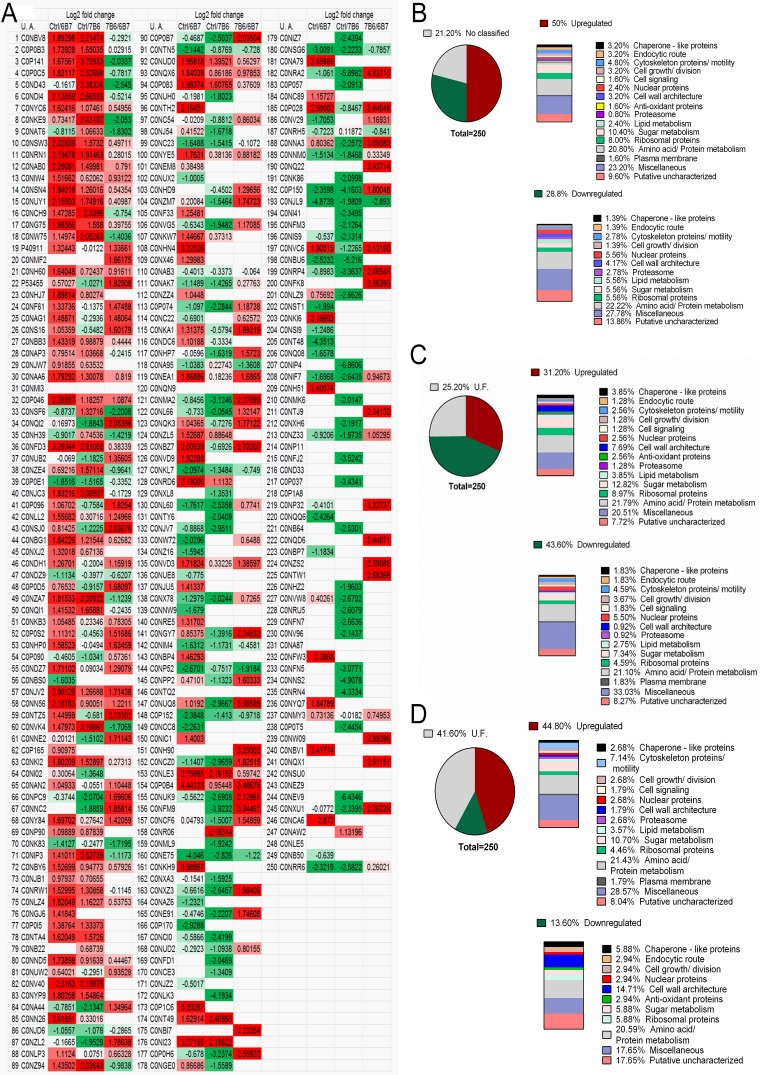
Ranking of differentially abundant proteins in extracellular vesicles after treatment of *H. capsulatum* yeast cells with MAb 6B7 or 7B6 in relation to vesicles isolated from untreated control yeast cells. (A) Global profile of proteins present in vesicles after treatment with MAb 6B7 or 7B6 in relation to untreated control vesicles. Red, upregulated proteins; green, downregulated proteins. (B, C) Proteins down- or upregulated after treatment with MAb 6B7 (B) or 7B6 (C). (D) Comparison of regulated proteins in vesicles isolated from yeast cells treated with MAb 7B6 or 6B7. U.A., UniProt accession number; U.F. (unknown function), nonabundant proteins.

Treatment of *H. capsulatum* cells with MAb 6B7 or 7B6 changed the profile of proteins in the vesicles in relation to that in untreated control vesicles (Fig. 5A; see Table S5 in the supplemental material). After treatment with MAb 6B7, 46.8% of the proteins were reduced in quantity and about half (45.8%) were increased (Fig. 5B). Although MAb 7B6 treatment also changed the expression profile, there were increases in 31.2% of the proteins and 43.6% were reduced (Fig. 5C). Treatment with MAb 6B7 most significantly reduced proteins associated with amino acid/protein metabolism (22.2%), followed by sugar metabolism, nuclear, ribosomal, and lipid metabolism proteins (5.6%) and cell wall architecture proteins (4.2%). Among the proteins with higher abundance after treatment with MAb 6B7, 20.8% were associated with amino acid/protein metabolism, 10.4% were associated with sugar metabolism, and 8% were ribosomal proteins (Fig. 5B). Analyzing the set of proteins altered after treatment with MAb 7B6, we determined that the quantities were reduced 21.1% for proteins involved in amino acid/protein metabolism, 7.3% for sugar metabolism proteins, and 5.5% for nuclear proteins (Fig. 5C), and the quantities were increased 21.8% for proteins involved in amino acid/protein metabolism, 12.8% for sugar metabolism proteins, and 9% for ribosomal proteins (Fig. 5C). We also compared the abundance of proteins in vesicles isolated from yeast cells after treatment with MAb 7B6 in relation to that of proteins in vesicles from cells treated with MAb 6B7. The analysis showed that most proteins were increased (44.8%) in the MAb 7B6 vesicles compared to the MAb 6B7 vesicles, with proteins associated with amino acid/protein metabolism having the most abundance (21.4%), followed by sugar metabolism proteins (10.7%) and cytoskeleton proteins (7.1%) (Fig. 5D). This comparison also showed that treatment with MAb 7B6 reduced more proteins, with 20.6% of these proteins being associated with amino acid/protein metabolism and 14.7% being cell wall architecture-related proteins. Interestingly proteins related to cell wall architecture, such as cell wall remodeling protein, cell wall synthesis protein, and 1,3-β-glucanosyltransferase, were decreased in vesicles isolated from yeasts treated with MAb 6B7 and increased in MAb 7B6-treated vesicles. Furthermore, MAbs 6B7 and 7B6 changed the abundance of polyphenol oxidase and alkaline phosphatase enzymes in relation to that in untreated control vesicles, as both were increased according to proteomic analysis (see Table S5 in the supplemental material). Notably, alteration of the abundance of vesicle proteins also occurred after treatment with the Hsp60-binding control MAb 12D3, where 50% of the proteins were increased and 32.4% were reduced (see Table S5 in the supplemental material). MAb 12D3 produces biological and protective responses similar to those of MAb 6B7 (8) and was thus utilized to support our evidence that protective MAbs produce differential loading of vesicles compared to the one nonprotective MAb to Hsp60 that we currently have.

### Fungal extracellular vesicle orthologues.

**FIG 6:**
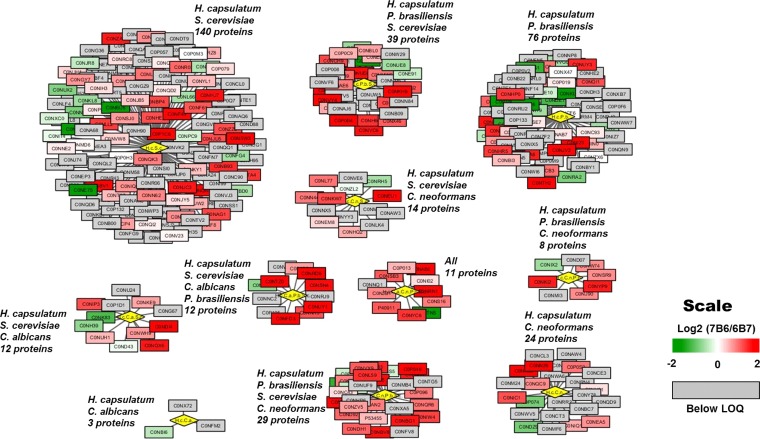
Analysis of orthologous proteins found in different fungal extracellular vesicles. Each subnetwork is rooted by the common species upon which the orthologous proteins were identified, represented by the yellow diamonds. Each subnetwork contains only proteins exclusively common to those species. The colors of the orthologous proteins (rectangles) represent differential abundances in extracellular vesicles treated with MAb 7B6 or 6B7. The orthologous proteins in gray were below the limit of quantification (LOQ).

To understand whether or not 6B7 and 7B6 MAb treatments would differentially affect conserved functions, we additionally compared *H. capsulatum* extracellular vesicle proteins with orthologous proteins carried by vesicles from *P. brasiliensis*, *C. neoformans*, *Saccharomyces cerevisiae*, and *C. albicans* (Fig. 6). We assumed that the more conserved the functions is, the more commonly would protein orthologues related to these functions be found in more different species. The network layout was according to proteins commonly found in different species, with each subnetwork containing only proteins shared by common species (represented by yellow diamonds). Each rectangle represents a protein orthologue and is colored according to its differential abundance in vesicles released by cells treated with different MAbs. A total of 11 common protein orthologues were found in all of the species analyzed, and most of them were upregulated after treatment with MAb 7B6 (Fig. 6). Comparison of the etiologic agents of pulmonary fungal infections (*H. capsulatum*, *P. brasiliensis*, and *C. neoformans*) showed that 48 proteins were common to all three of these fungal species (see subnetworks of all species; *H. capsulatum*, *P. brasiliensis*, *S. cerevisiae*, and *C. neoformans*; and *H. capsulatum*, *P. brasiliensis*, and *C. neoformans*) (Fig. 6). In this case again, the protein orthologues common to the pulmonary pathogen species were differentially abundant in extracellular vesicles derived from cells treated with MAbs 6B7 and 7B6, reinforcing the idea that these antibodies differentially regulate conserved fungal pathways although they bear the same epitope.

## DISCUSSION

We previously demonstrated that opsonization of *H. capsulatum* cells with Hsp60-binding MAbs 6B7 (IgG1, protective antibody) and 7B6 (IgG2b, nonprotective antibody) significantly altered their phagocytosis rate and survival within macrophages, as well as modified the course of infection in a murine disease model (9). In the present work, we observed that treatment of *H. capsulatum* cells with these MAbs also changed the size of the extracellular vesicles produced by the fungus. This study builds upon our prior description of extracellular vesicle production by *H. capsulatum* (15) and the information regarding vesicle production by other fungi by demonstrating that treatment with different MAbs significantly alters the size and content of these biologically important vesicles. Hence, this is a new role for antibodies in fungal pathobiology. Furthermore, the finding adds another dimension to the observation that some antibodies can have direct effects on the physiology of microbes (21, 22).

Fungal cells produce a heterogeneous population of extracellular vesicles that vary notably in size and content (23). Our analysis of the protein contents of vesicles isolated from *H. capsulatum* cells treated with or without MAb 6B7 or 7B6 reveals that MAb exposure significantly increases the protein loading of vesicles. Treated vesicles are larger and contain more proteins than untreated control vesicles. Thus, opsonization with these MAbs appears to change the quality and quantity of vesicle cargo loading.

The binding of MAbs to the surface of a microorganism can modify the complex relationship between a host and a pathogen (9). Interestingly, our results reveal that treatment of *H. capsulatum* with MAb 6B7 or 7B6 results in differences in the activities of phosphatase, laccase, and catalase in vesicles, suggesting that these antibodies modulate the production, trafficking, and release into the extracellular space of important fungal virulence factors. The alteration of several proteins concomitantly is important, as modification of single proteins may not significantly impact pathogenicity. For example, the loss of the catalase CatB has no deleterious effect on *Histoplasma* virulence *in vivo* or *in vitro* (23), indicating that several enzymes are involved in the protection of the fungus from reactive oxygen species. Laccase catalyzes melanin synthesis, and the resultant pigment protects the fungal cells from oxidative stress, as well as from phagocytosis by macrophages. The reduction of laccase activity in vesicles from yeast cells treated with MAb 6B7 is an example of a specific factor whose presence is required for virulence, as best demonstrated in *C. neoformans* (24, 25), where smaller alterations in activity may translate to significant biological differences.

Proteomic analysis of the extracellular vesicles revealed a large and complex composition of proteins with diverse biological functions such as cell growth and signalization; protein, lipid, and sugar metabolism; cell wall architecture; the endocytic route; and antioxidant proteins. Interestingly, in contrast to the enzymatic assays, the proteomic analysis did not show differences in phosphatase, laccase, or catalase between the groups examined. This discrepancy could be impacted by several factors, such as (i) low sensitivity in the measurement of all cargo proteins, (ii) the possibility that the proteins identified are only a small fraction of the total proteins found in the vesicles, and (iii) an effect of antibody treatment (11, 17). As described by Albuquerque et al. (15) and Holbrook et al. (26), our data also show the presence of extracellular proteins involved in cell wall assembly (e.g., 1,3-β-glucanosyltransferase), and changes in the membrane environment could be involved in the reduction of enzymatic activity. In addition, there were also changes in the antioxidant proteins (e.g., a thiol-specific antioxidant protein) and chaperone and nucleus-associated proteins such as Hsp70 (15, 27).

The abundance of the same set of proteins in the vesicles was differentially modified, depending upon the MAb used to treat the cells, indicating that these MAbs specifically change the total proteins and their profile of abundance in the extracellular vesicles. Under all of the conditions evaluated, amino acids/proteins involved in metabolism were the most abundant proteins in the vesicles, suggesting that the MAbs profoundly impact the metabolism and transport of proteins (28). However, some protein changes were detected only upon treatment with one isotype of MAb, suggesting specific alterations in fungal physiology. For instance, levels of saccharopine dehydrogenase and oxysterol-binding protein were modified only upon MAb 6B7 treatment. The former protein is involved in lysine metabolism and was found be sensitive to decreasing iron levels (29). This modulation of metabolism might be advantageous for the survival of *H. capsulatum* cells in a nutritionally restricted environment such as the immune cell milieu (29). The latter, oxysterol-binding, protein plays a role in ergosterol synthesis, potentially impacting antifungal targets and membrane stability (30). In addition, downregulation of β-glucan proteins (such as 1,3-β-glucanosyltransferase) after MAb 6B7 treatment indicates modification of the synthesis of β-glucan, a structural constituent of the fungal cell wall and a target for host immune system cells (19). Indeed, changes in the profile of abundance of proteins related to protein metabolism and sterol and β-glucan synthesis suggests important modifications of the *H. capsulatum* cell wall upon MAb 6B7 treatment. Thus, these alterations may impact fungal virulence, the immune response, and treatment with antifungal agents that target sterol (1, 19).

Treatment with disease-enhancing MAb 7B6 induced more alterations in the magnitude of protein abundance than did treatment with protective MAb 6B7. Increases in the abundance of sugar metabolism proteins that were upregulated (such as malate dehydrogenase and aconitase) suggests that opsonization with MAb 7B6 enhances energy acquisition. This change was accompanied by an increase in the abundance of amino acid/proteins involved in metabolism that were upregulated, which is consistent with augmentation of protein metabolism. In addition, the increased abundance of cytoskeleton protein/motility-associated proteins indicates that opsonization with MAb 7B6 also enhances intracellular motility. Thus, the interaction of MAb 7B6 with cell surface Hsp60 may lead to a protective adaptation of fungal cells to stress responses and consequently change the loading of proteins in the secreted vesicles, enhancing cellular resistance to host defenses (9, 19).

The fungal species analyzed are from the phylum *Ascomycota*, and orthologue analyses demonstrate that they release common extracellular components to deliver diverse macromolecules to the extracellular space (13, 22). Heat shock proteins, the most highly evolutionarily conserved proteins, were found to be upregulated in all of the fungal species tested and in *H. capsulatum* vesicles after treatment with MAb 7B6. Heat shock proteins are generally produced in response to challenging conditions (e.g., high temperature, oxidative stress, radiation, and inflammation). The binding of MAb 7B6 to *H. capsulatum* appears to induce a stress situation in the yeast that may prime it for more effective survival of host-pathogen interactions. As previously shown, the proteins in *H. capsulatum* vesicles had many similarities to proteins identified in vesicles of *Saccharomyces cerevisiae* (15, 31) and treatment with MAbs 6B7 and 7B6 did not change the profile of the similar proteins, suggesting that regulation of the concentrations of many of these orthologous proteins may not be essential for survival *in vivo*. Interestingly among the etiologic agents of pulmonary infections, *H. capsulatum* had more proteins in common with *P. brasiliensis* than *C. neoformans*, suggesting that these endemic fungi share more characteristics than dimorphism, an infection route, and the capacity to cause disease in immunocompetent individuals.

In conclusion, our results reveal that treatment with MAbs 6B7 and 7B6 changes vesicle size and increases the protein loading of the vesicles. We found that urease, phosphatase, laccase, and catalase were present in vesicles isolated from yeast cells grown with or without these MAbs, confirming that *H. capsulatum* vesicles are involved in the delivery of virulence factors to the extracellular space and demonstrating that binding by MAbs can modify the quantity of biologically relevant proteins in vesicles. This finding is clearly in line with prior studies of *C. neoformans* showing that antibody binding can directly impact gene regulation and fungal metabolism (21, 32). Finally, analysis of orthologous proteins showed that different ascomycetes produce similar structures in extracellular supernatants with similar proteins in their milieu, corroborating the idea that vesicles are important effectors involved in the communication between intra- and extracellular spaces. Hence, further studies of modified vesicle production and function in the setting of antibodies may provide insights into novel approaches to modifying the pathobiology of these potentially lethal pathogens.

## MATERIALS AND METHODS

### Strain and media.

*H. capsulatum* strain ATCC G217B was cultivated in Ham’s F12 medium (supplemented with glucose [18.2 g/liter], glutamic acid [1 g/liter], HEPES [6 g/liter], cysteine [8.4 mg/liter], and a penicillin-streptomycin solution [1%]) at 37°C in a rotary shaker (150 rpm) for 7 days (33).

### MAb production.

The generation of MAbs in ascites fluid was approved by the Albert Einstein College of Medicine Institutional Animal Care and Use Committee. Briefly, IgG1 (6B7) and IgG2b (7B6) MAbs were produced by injecting 10^7^ hybridoma cells into the peritoneal cavities of ex-breeder BALB/c female mice (National Cancer Institute) that had previously been primed with Pristane (Sigma-Aldrich). The concentration of MAbs in the ascites fluid was determined by enzyme-linked immunosorbent assay with IgG1 and IgG2b standards at known concentrations (34). The same procedures were performed to generate MAb 12D3 (IgG2a), which binds a different region of *H. capsulatum* Hsp60 (8) and was used as a positive control in the proteomic analyses.

### Vesicle purification.

Vesicles were purified according to the protocol described by Rodrigues et al. (35), with minor modifications. *H. capsulatum* yeast cells (2.5 × 10^6^/ml in a volume of 30 ml) were incubated with MAb 6B7, 7B6, or 12D3 at 6 µg/ml. To maintain the log phase, 10 ml of fresh medium was added to the cells every 48 h (final volume of 50 ml). After 7 days of growth, the yeast cells were removed by centrifugation at 3,000 rpm for 10 min at 4°C and then filtered with a 0.45-µm-pore-size filter (17). Cell-free supernatant was concentrated in an Amicon ultrafiltration system with a membrane with a 100-kDa cutoff. After filtration, the membrane was washed with filtered phosphate-buffered saline (PBS) to collect any remaining vesicles on the membrane surface. The collected vesicles were further centrifuged at 152.813 × *g* (60,000 rpm, with a TLA 100.3 rotor in a Beckman Coulter ultracentrifuge) for 1 h at 4°C. The supernatant was removed, and the pellets were suspended in 0.1 ml of filtered PBS, combined, and submitted to repeat ultracentrifugation. For proteomic analysis, the pellets were used, whereas vesicles were suspended in 0.5 ml of filtered PBS containing protease inhibitor cocktail (Roche) for DLS analysis, quantification, and enzymatic activity determination. All experiments were performed in duplicate.

### Analysis of extracellular vesicle size by DLS.

The size distributions of extracellular vesicles suspended in PBS supplemented with a protein inhibitor cocktail (Roche) were measured by quasielastic light scattering in a 90Plus/BI-MAS multiangle particle sizing analyzer (Brookhaven Instruments). In solution, vesicles undergo Brownian motion that, after illumination by monochromatic laser, produces light scattering fluctuations (i.e., DLS) that provide information about size distribution (17). All experiments were performed in duplicate.

### Protein and sterol quantification.

Protein quantification was performed with Bradford reagent (Bio-Rad, Richmond, CA) by NanoDrop technology (ND-1000 spectrophotometer; Thermo Scientific). Sterol quantification was performed with an Amplex Red kit (Life Technologies).

### Enzyme activity.

To detect urease, phosphatase, and laccase activities in the vesicles, Vesicle suspension volumes of 30 µl with a protein concentration of 10 µg/ml were aliquoted to a 96-well plate (17). One hundred microliters of each enzyme reaction solution was added, and plates were stored at 37°C while protected from the light for 16 h and then read with a spectrophotometer (BioTek). Urease activity was evaluated with an enzyme reaction mixture containing 1% peptone, 0.1% dextrose, 0.5% NaCl, 0.2% KH_2_PO_4_, 2% urea, and 0.0012% phenol red. The plate was read at 540 nm. To evaluate phosphatase activity, the reaction buffer was prepared with *p*-nitrophenylphosphate at 1 mg/ml of 100 mM sodium acetate solution. The reaction was read at 405 nm. For laccase evaluation, the solution was prepared with 12.5 mM of l-3,4-dihydroxyphenylalanine in PBS and the plates were read at 450 nm. Finally, catalase activity (with a protein concentration of 10 µg/ml) was evaluated with a catalase assay kit (Cayman Chemical).

### Sample preparation for proteomic analysis.

*H. capsulatum* extracellular vesicle pellets, prepared in biological replicates, were suspended in 100 µl of 50 mM NH_4_HCO_3_ containing 5 mM dithiothreitol (DTT) and 8 M urea and incubated for 15 min at 37°C in order to reduce disulfide bonds. Free thiol groups were alkylated by adding iodoacetamide (IAA) to a final concentration of 10 mM and incubating the mixture for 30 min at room temperature. DTT was added to a final concentration of 20 mM to terminate the reaction. Samples were then diluted 8-fold with 50 mM NH_4_HCO_3_, and CaCl_2_ was added to a final concentration of 1 mM. Proteins were digested overnight at 37°C with 2 µg of trypsin. Reagents and salts were removed from the samples with solid-phase extraction C_18_ spin columns (Ultramicrospin columns, C_18_, 3- to 30-µg capacity; Nest Group). Briefly, 100 µl of each solution was loaded and the column was spun for 1 min at 1,000 rpm. The column was washed twice with 100% methanol and then washed twice with 0.1% trifluoroacetic acid (TFA). Samples were then loaded and washed four times with 5% acetonitrile (ACN) containing 0.1% TFA before elution with 80% ACN–0.1% TFA. The resulting peptides were dried in a vacuum centrifuge and suspended in a 0.1% formic acid (FA) solution for liquid chromatography-tandem mass spectrometry (MS) analysis.

### Global quantitative proteomic analysis.

Peptides were loaded into a C_18_ trap column (200 µm by 0.5 mm, ChromXP C_18_-CL, 3 µm, 120 Å; Eksigent), and separation was carried out in a capillary C_18_ column (75 µm by 15 cm, ChromXP C_18_-CL, 3 µm, 120 Å) connected to a nanoHPLC system (Ekspert nanoLC 400; Eksigent). Elution was performed with the following gradient: 1 min in 5% solvent B (solvent A, 0.1% FA; solvent B, 80% ACN–0.1% FA), 5 to 35% solvent B in 60 min, 35 to 80% solvent B in 1 min, 6 min in 80% solvent B, 80 to 5% solvent B in 1 min, and holding at 5% solvent B for 11 min. The flow rate was constant at 200 nl/min over the whole gradient. Eluting peptides were directly analyzed in an electrospray ionization mass spectrometer (5600 TripleTOF; AB Sciex). Full MS spectra were collected in a range of 400 to 2,000 *m*/*z*, and the 50 most intense parent ions were submitted to fragmentation for 50 ms with rolling-collision energy.

Peptides were identified by searching tandem mass spectra against a sequence database containing the *H. capsulatum* complete proteome set from the UniProt Knowledge Base and common contaminant sequences (9,465 total sequences) with the Paragon tool of the Protein Pilot software (AB Sciex). For database searches, trypsin digestion, cysteine residue alkylation with IAA, and biological modifications were considered as factors. Peptides were filtered with a confidence score of >95, which resulted in a false-discovery rate (FDR) of <2% at the protein level on the basis of the reserve sequence database approach.

Peptide and protein quantification was done by extracting peak areas of identified peptides with Skyline (Maclean). For differential-expression (DE) analysis, we used the hierarchical Bayesian model proposed by Wei and Li (36) with the mapDIA software (http://mapdia.sourceforge.net). More importantly, mapDIA allows the analysis of repeated measurements in quantitative proteomic data analysis, such as intensity data from multiple peptides within a protein or transition intensity data acquired from data-independent acquisition MS. We used the peptide isotopic intensity data (M, M + 1, M + 2) as repeated measures of peptide abundance in mapDIA. In the model, two possible probability models of intensity data are proposed for each compound, namely, a DE model and a non-DE model, and the posterior probability of DE is calculated and these scores are used to derive the FDRs for the selection of DE proteins (37).

### Statistical analyses.

Statistical analyses were performed by one-way analysis of variance or the Newman-Keuls multiple-comparison test with GraphPad Prism software, depending on the data.
