# Supplementary material for: Antibody Binding Alters the Characteristics and Contents of Extracellular Vesicles Released by Histoplasma capsulatum
Source: mSphere. 2016 Mar 30;1(2):e00085-15. doi: 10.1128/mSphere.00085-15 (PMC4894687; doi:10.1128/mSphere.00085-15)
Supplement: Table S1 [file sph002162053st1.docx]

**Supplemental Table 1:** Total protein analysis and sterol content quantification in vesicles from *H. capsulatum* yeast cells with or without treatment with mAb 6B7 or 7B6.

| **Total protein (μg/mL)** | | |
| --- | --- | --- |
| **Control** | **6B7** | **7B6** |
| 52.2 | 457.2^**^ | 485.0^**^ |
| 66.5 | 418.2^**^ | 553.0^**^ |
| **Sterol content (μg/mL)** | | |
| **Control** | **6B7** | **7B6** |
| 5.40 | 9.10 | 4.71 |
| 7.33 | 5.99 | 6.95 |
| **Ratio (total protein/sterol content)** | | |
| **Control** | **6B7** | **7B6** |
| 8.20 | 60.62^**^ | 83.19^**##^ |
| 10.45 | 55.45^**^ | 94.85^**##^ |

**Compared to untreated control. ^##^Compared to 6B7 mAb. **^,##^ p < 0.05.
